# Promoting rural residents' participation in clinical trials: clinical trials basics programming and training for rural public librarians

**DOI:** 10.5195/jmla.2023.1650

**Published:** 2023-07-10

**Authors:** Dana L. Ladd, Jackson C. Wright

**Affiliations:** 1 dlladd@vcu.edu, Health and Wellness Librarian, Virginia Commonwealth University, Richmond, VA.; 2 wrightjc@vcu.edu, Health and Wellness Library Assistant, Virginia Commonwealth University, Richmond, VA.

**Keywords:** Outreach, public library, consumer health library, clinical trials, health literacy, consumer health

## Abstract

**Background:**

Having diverse representation in clinical trial participation is important. Historically, rural residents have been underrepresented in clinical trial research. Public librarians have an opportunity to promote clinical trial participation among rural residents by offering consumer health information services that help patrons to understand what clinical trials are and how they can find relevant clinical trials.

**Case Presentation:**

A consumer health library and a clinical trial center located at a large academic medical center collaborated to provide clinical trial information programming to rural public libraries. The group was awarded a Network of the National Library of Medicine (NNLM) Community Outreach Grant and was able to plan, develop, promote, and implement programs including training workshops, a speaker event, and a book discussion to rural public librarians.

**Discussion:**

Marketing the programs to rural public libraries was difficult and many barriers were encountered. Though registration and subsequent participation were low, participants expressed interest and gratitude for the programs. For any future programs targeting this population, further strategies will need to be implemented to ensure increased registrations and attendees.

## BACKGROUND

Rural and minority communities have been historically underrepresented in clinical trial research. Without the contribution of a diverse group of research participants, knowledge generated by health research could have minimal application. Public libraries can improve the health literacy of underrepresented populations by providing these groups with reliable consumer-level information about clinical trials. Public librarians are highly skilled in providing general reference services to the public but may feel unprepared to answer health-related questions including those about clinical trials.

In this paper, we describe a collaboration between Virginia Commonwealth University (VCU) Health and Wellness Library and the VCU Wright Center for Clinical and Translational Research that aimed to provide public librarians in Virginia with the skills to increase the health literacy of community members regarding clinical trials.

Clinical trials test the efficacy and safety of new medical advances and approaches on human subjects. There are various types of clinical trials in which subjects can enroll, including prevention, screening, diagnostics, treatment, behavioral, and quality of life trials. Certain populations have been historically underrepresented in clinical trials. Research suggests that rural and minority communities participate in research at lower numbers because of cultural sensitivity, geographic challenges (e.g. distance and isolation), misperceptions of research, and lack of opportunities to participate [1,2]. The United States Food and Drug Administration (FDA) states, “this is a concern because people of different ages, races, and ethnicities may react differently to certain medical products.” [3]

Clinical trial participation in rural medically underserved areas is low. Trial investigator respondents report difficulty finding rural residents to participate in clinical research studies [4, 5]. Identified barriers for rural resident participation in clinical trials include: time and effort to participate, lack of health insurance and potential costs, medical providers unaware of clinical trials, poor access to medical care, lack of convenient or affordable transportation, distance and isolation, cognitive barriers, limited knowledge and awareness of clinical trials, clinical trial misperceptions, distrust, and historical abuses [6]. Additionally, many may be unfamiliar with sites for locating relevant clinical trials such as ClinicalTrials.gov. Those living in rural areas have less income and higher poverty rates, a lower educational level, and a lower health literacy level than those in urban areas. Rural areas are also often medically underserved and rural residents often do not have access to health care providers, clinics, and hospitals. Many rural residents also lack employer-provided health insurance.

For those living in rural areas, these factors create a significant impact on health, and consequently, rural residents experience significant health disparities. Those living in rural areas experience higher rates of chronic health conditions and disabilities, such as obesity and high blood pressure, compared to residents in urban areas. Rural residents also experience higher mortality rates and a lower life expectancy and are more likely to die of heart disease, cancer, unintentional injury, chronic lower respiratory disease, and stroke than those living in metro areas [7, 8]. Rural residents are the very population who may benefit greatly from access to and participation in clinical trials.

Public librarians often feel unprepared to answer their patrons' consumer health questions due to lack of knowledge and training about reliable consumer health information resources [9, 10]. Patrons do, however, view their local public libraries as trusted sources of information [11, 12]. Training public librarians to increase the health literacy skills of the community members they serve is essential to ensure that patrons have access to reliable health information written at a level they can understand, and which they can use to make informed and appropriate decisions regarding their health. Health literacy partnerships with public libraries have allowed opportunities for public librarians to learn more about health literacy and finding consumer health information for their patrons [13, 14, 15].

Patrons with strong health literacy skills can learn about and understand clinical trials, make informed decisions about participation in clinical trials, and will be able to locate appropriate clinical trials for which they are eligible.

## CASE PRESENTATION

The Virginia Commonwealth University (VCU) Medical Center Health and Wellness Library collaborated with the VCU Wright Center for Clinical and Translational Research and applied for the Network of the National Library of Medicine (NNLM) Community Outreach grant. The collaboration was awarded $20,000 to develop and conduct clinical trial programming for rural public librarians. Over several weeks in mid-spring 2022, we offered a clinical trials/consumer health information finding workshop (5 sessions of the same program); two one-hour book discussion sessions; and a one and half hour speaker event held via Zoom.

### Clinical Trials and Consumer Health Information Training Workshop

The workshop was designed to give library employees the confidence to search for patron-requested health information and included information on the basics of clinical trials, suggested methods for finding reliable consumer health information, and interactive demonstrations of MedlinePlus and ClinicalTrials.gov. The same one-hour session was offered on multiple dates and times to facilitate public librarian attendance.

The Wright Center's senior hub research capacity administrator developed and taught the clinical trials basics portion of the workshop. The Health and Wellness Library Librarian developed and taught the consumer health information portion of the workshop and demonstrated how to use MedlinePlus and Clinicaltrials.gov.

The first 30 minutes of the workshop introduced clinical trials and highlighted the following topics:

- Overview of the Center for Clinical and Translational Research
- Clinical trials basics
- What is a clinical trial?
- Why are clinical trials important?
- Historical inequities in clinical trials
- Types of clinical trials
- Informed consent / Rights and protections

The second half of the workshop was on finding reliable consumer health information and clinical trials. This thirty-minute session comprised the following topics:

- Introduction to the Health and Wellness Library
- Evaluating online health information
- Demonstration of MedlinePlus.gov
- Demonstration of Clinicaltrials.gov

Following the workshop, public librarians had the opportunity to ask questions about clinical trials and/or finding reliable consumer health information.

### Speaker Event

The library and the Wright Center hosted a one-and-a-half-hour speaker event via Zoom to promote clinical trials participation. Cancer survivor, patient advocate, and international keynote speaker e-Patient Dave (Dave deBronkhart), a stage IV kidney cancer survivor, shared his cancer journey including advocating for his own health and participating in a clinical trial which ultimately saved his life. Through his engaging talk e-Patient Dave encouraged patients to advocate for their own care and provided tips on how to become a more empowered patient. Concluding e-Patient Dave's talk was a facilitated question and answer session. In addition to public librarians, this event was open to anyone and included a diverse audience of public librarians, academic health sciences librarians, health care providers, patients, and community members/public.

### Book Discussion

The library selected an appealing medical memoir that focuses on clinical trials (from a list developed by the library staff): *A Series of Catastrophes and Miracles: A True Story of Love, Science, and Cancer* by Mary Elizabeth Williams. We held two book discussion sessions of the book, allowing public librarians a choice of dates and times. Each book discussion was one hour long and open to all public libraries' staff. All three of the project planners developed the discussion questions, attended, and moderated the book discussion group. The project planners were available to answer any questions about health literacy/consumer health information and clinical trials questions that arose during the book discussion and promoted library and clinical trial resources. Everyone who registered for the book discussion sessions was sent a free copy of the book via mail.

### Marketing

The target population of this project is rural public libraries surrounding and near Richmond, Virginia. We used the Rural Health Information Hub (RHIhub) to identify rural designated areas and if those areas were deemed medically underserved, as well as their medical shortage designations. We used the University of North Carolina Health Literacy data map (US Health Literacy Scores (unc.edu) to determine the health literacy levels of residents in these counties. We indicated if these counties have areas with high percentages of health literacy levels in the lowest state quartiles (level 1 and level 2- basic or below basic). Libraries that we targeted in our marketing campaign were those that are in areas designated as rural and as having low health literacy. We initially targeted 37 rural public libraries that are within a one-hundred-mile radius of our library.

We created flyers and short messaging about the programs. We then emailed these program descriptions, dates, and registration links to 37 rural public libraries. Due to low response, we also mailed flyers and placed phone calls to libraries. We decided to expand the invitation to all 95 public libraries within the state and then advertised the programs via the Virginia Library listserv in order to reach more librarians.

### Program Evaluation

In order to assess the program's impact, we collected the number of registrations, number of attendees, feedback from evaluation questions, and feedback in the chat sessions of the programs. We used the NNLM program evaluation questions and input those questions into QuestionPro. QuestionPro is an online survey software tool to which library subscribes. The link to complete the evaluation was sent out via email one hour following each program.

### Clinical Trials and Consumer Health Information Training Workshop

Five Clinical Trials Workshop sessions were offered. A total of twelve public librarians attended the workshops. Informal feedback via Zoom chat was positive, with participants expressing thanks and appreciation for the session. A library director from a small rural public library who was unable to participate in the workshop contacted us to request a training session for library staff. A formal evaluation questionnaire was sent out to participants following the program. Of the twelve librarians completing the workshop only two completed the evaluation. Though the two responses were generally positive given the low response rate, it is difficult to assess the impact of the workshops. Verbal feedback following the workshops was very positive. Participants expressed appreciation for the workshop and expressed that the workshop was useful. In the formal evaluation survey, both respondents reported that they were introduced to a resource they had never used before, learned a new skill they plan to use in the future, and that the workshop improved their ability to use a resource they already use. One reported “strongly agree” and the other “somewhat agree” to the statement that the workshop improved their ability to find useful health information. Both respondents reported an increase in knowledge of and comfort level using both MedlinePlus.gov and ClinicalTrials.gov

### Speaker Event

A speaker event was held at noon via Zoom. A total of sixty people attended. Four identified as a public librarian or registered with a public library email address when registering for the program. Other attendees were librarians from hospital and academic institutions, healthcare providers, and members of the public. Informal feedback via Zoom chat was positive, with participants expressing thanks and appreciation for the session.

### Book Discussion

We provided two book discussion sessions of the memoir, A Series of Catastrophes and Miracles: A True Story of Love, Science, and Cancer. One session was conducted in the morning and another in the afternoon on different days. Participants could select a convenient time to attend. Seven people registered for the first session, and of those seven, four people attended the session. Two people signed up for session two and two people attended (however one may have had connectivity issues and came in/out). We mailed out copies of the book to all registered.

**Table 1:** Speaker event evaluation results

n=19	“Yes”	“No”	N/A			
Were introduced to at least one health information resource or tool that they had never used before	84.21% n=16	15.79% n=3			
Learned a new skill in the training session that they plan to use in the future	84.21% n=16	15.79% n=3			
Improved their ability to apply a resource they already use	84.21% n=16	0	15.79% n=3		
	Very Satisfied	Satisfied	Neutral	Somewhat Disagree	Strongly Disagree
Were satisfied with the program	94.74% n=18	5.26% n=1	-	-	-
	Strongly Agree	Somewhat Agree	Neutral	Somewhat Disagree	Strongly Disagree
Improved their ability to find useful health information online	68.42% n=13	26.32% n=5	5.26% n=1	-	-
	Most Likely	Somewhat Likely	Neutral	Somewhat Unlikely	Least Likely
Likely to tell others about a resource or tool that they learned about in the workshop	68.42% n=13	26.32% n=5	5.26% n=1	-	-

Verbal and chat feedback following the program were very positive. Verbal feedback was positive following both book discussions and attendees reported gratitude for us sending them copies of the book that we were discussing. Only two participants completed the evaluation form sent out following the program. Both respondents reported being introduced to at least one health information resource they had never heard of, learned a new skill, and improved their ability to apply a resource they already use.

## DISCUSSION

Despite the low attendance rates, this project was beneficial to those who attended, and we were able to have a small but positive impact on rural public libraries. Public librarians and staff who participated in this project indicated having a clearer understanding of what clinical trials are, how to locate clinical trials, and how to find reliable consumer health information. They can provide support to their library patrons and improve health literacy skills of their patrons so that patrons can make informed decisions about their health and participation in clinical trials. Participants expressed their gratitude following the programs and indicated their willingness to share information learned with their colleagues. Additionally, following the program, a library director from a very small rural library in the state reached out and expressed interest in having us conduct a clinical trial and consumer health information training session for her staff.

One of the greatest barriers encountered during the project was the difficulty in promoting the events directly to rural public librarians and staff, which limited the program's overall impact. One of the contributors to this barrier was the difficulty in reaching the target audience via email, phone, and mail. Most of the email addresses to contact libraries on public-facing library web pages are generic emails or contact forms. We were uncertain if the emails actually reached anyone. We were also unsure whether someone in the library did receive the invitation, and that it was then disseminated among library staff. Also, the fliers we attached to emails may have been filtered by many library systems' email accounts. Even though the library listserv reached many librarians in the state, not all librarians/library staff are members of the listserv.

Another contributing factor to low registration was the staff shortages that many libraries are facing due to the impact of COVID-19. Many rural public libraries may have limited staff and may have difficulty attending workshops during operating hours when service points require staffing. Staff shortages contribute to increased workloads for remaining staff. Another contributing factor to low attendance could possibly have been burnout due to the COVID-19 pandemic and ongoing health misinformation. A recent survey published in Public Libraries stated that 57% of public librarians surveyed reported experiencing burnout in the workplace as a result of the pandemic [16].

One valuable lesson learned is that if reaching out to rural public libraries, be willing to not only try various strategies to promote programming, including email, phone calls, fax, mailing, and use of any appropriate listservs, but also speak with public librarians/library staff to identify any barriers to participation on their end so you can proactively work to overcome those barriers.

The program evaluations did not measure quantifiable learning, and though the limited number of responses were overall positive, they did not indicate whether the information and tools taught would actually be used by library staff. There was no long-term follow-up to measure use of clinical trial information with library patrons.

Due to funding limitations, there are no future plans for additional clinical trial programming aimed at public librarians at this time. We invited each of the attendees to contact us if they had any additional questions about finding consumer health information or about clinical trials. We will continue to be a point of reference for rural public librarians who have questions about clinical trials. One rural library whose staff was unable to attend the workshops reached out to us expressing interest in conducting a workshop on finding consumer health information. For any future programs targeting this population, further strategies will need to be implemented to ensure increased registrations and attendees.

## Data Availability

All variables are represented in the text of the manuscript, with none excluded or suppressed.
